# Effect of Hydrogen Peroxide, Ytterbium Fiber Laser, and Phthalocyanine-Mediated Low-Level Laser Therapy on Surface Characteristics, Flexural Strength, and Push-out Bond Strength of Glass Fiber Posts to Root Dentin

**DOI:** 10.12669/pjms.42.7.15915

**Published:** 2026-07

**Authors:** Waleed M.S Alqahtani, Abdelaziz Almasry, Hassan Alsubhi, Yasmeen Bandar Alotaibi, Nasser Hussein Shaheen

**Affiliations:** 1 Waleed M.S Alqahtani King Khalid University, Department of Prosthetic Dental Sciences, College of Dentistry, Saudi Arabia; 2 Abdelaziz Almasry King Saud University, Saudi Arabia; 3 Hassan Alsubhi Umm Al-Qura University, Saudi Arabia; 4 Yasmeen Bandar Alotaibi Department of Prosthetic Dental Sciences, College of Dentistry, Qassim University, Qassim, Saudi Arabia; 5 Nasser Hussein Shaheen Department of Restorative and Prosthetic Dental Sciences, College of Dentistry, Dar Al Uloom University, Riyadh, Saudi Arabia

**Keywords:** Hydrogen peroxide, Ytterbium fiber laser, Phthalocyanine, Self-Adhesive Resin cement

## Abstract

**Objective:**

Assessing the impact of different surface conditioners (Hydrogen peroxide (H_2_O_2_), Ytterbium fiber laser (YFL), Low-level laser therapy (LLLT)-Phthalocyanine (Pc) on the surface characterization, flexural strength (FS), surface roughness (R_a_) and push-out bond strength (POBS) of glass fiber post (GFP) bonded to root dentin using conventional resin cement (CRC) and self-adhesive resin cement (SARC).

**Methodology:**

This in-vitro study included eighty single-rooted mandibular premolar teeth, and subsequently, crowns were decoronated. Ethical approval was obtained from Dar Al Uloom University (COD/IRB/2025/91; Dated February 15, 2025). The study duration was six months. 120 GFP were allocated to four groups according to the conditioning protocol. Group-1 (No conditioning), Group-2 (H_2_O_2_), Group-3 (YFL), and Group-4 (1% Pc-LLLT). Scanning Electron Microscopy (SEM) was performed to assess the surface topography. R_a_ was measured using a profilometer. The three-point bending test was used to evaluate the flexural strength (FS). Root canal procedure was performed, followed by post space preparation. Twenty GFPs were distributed into two subgroups based on the bonding technique- Subgroup A: CRC via total etch (TE); Subgroup B: SARC*.*POBS and bond failure assessment were performed using a universal testing machine and a stereomicroscope*. Two-way ANOVA and Tukey post* hoc test were applied for all comparisons.

**Results:**

The highest R_a_ and lowest FS were seen in Group-2 (H_2_O_2_). The lowest R_a_ and the highest FS were observed in Group-1 (No conditioning) samples. The highest POBS was observed in the cervical third of Group-2A (H_2_O_2_+CRC). Conversely, the lowest bond strengths were observed in the apical section of Group-4A (1% Pc-LLLT+CRC).

**Conclusion:**

The yttrium fiber laser demonstrated satisfactory R_a_, FS, and POBS for the glass fiber post. Self-adhesive resin cement outperformed conventional luting cement.

## INTRODUCTION

Endodontic therapy is indicated in cases of deep carious lesions, dental trauma, excessive wear, or failure of prior restorative procedures. However, substantial loss of dental tissue following endodontic intervention typically compromises the tooth’s structural integrity, necessitating reliable restorative solutions that re-establish both aesthetics and function.^1^ In contemporary dental practice, post-and-core systems are the most widely adopted approach for rehabilitating endodontically treated teeth, ensuring adequate retention and structural continuity between the root and the definitive crown.^2^ Among the available options, glass fiber posts (GFPs) are increasingly favored over conventional cast metal posts, as their elastic modulus closely approximates that of dentin, thereby distributing functional stresses more favorably along the root and reducing the risk of catastrophic root fractures.^3^ Nevertheless, the predominant mode of GFP failure remains debonding at the post–resin cement interface, attributable to insufficient adhesion.

Various surface pretreatment protocols have been investigated to enhance the bond strength of GFPs to resin cement. Hydrogen peroxide (H_2_O_2_) is among the most commonly employed agents; it partially dissolves the epoxy resin matrix surrounding the glass fibers, thereby increasing surface roughness and facilitating micromechanical interlocking with resin cement.^4^ However, concerns persist regarding potential compromise of the post’s flexural strength and the generation of residual oxygen that may inhibit resin polymerization.^4^ Consequently, alternative surface conditioning strategies that preserve post integrity while enhancing adhesion warrant investigation.

Laser-assisted surface conditioning has recently emerged as a promising alternative. Pulsed ytterbium fiber laser (YFL) irradiation, operating at 1070 nm, modifies surfaces through thermomechanical ablation and has demonstrated efficacy in enhancing the roughness and adhesion of ceramic substrates such as zirconia.^5^,^6^ However, evidence regarding its effects on the surface roughness, flexural strength, and bond strength of GFPs remains limited. Concurrently, photodynamic therapy using phthalocyanine (Pc) as a photosensitizer, activated by low-level laser therapy (LLLT), has been explored in dentistry.^7^ Phthalocyanine exhibits high photostability, strong absorption within the therapeutic window (600–900 nm), and efficient generation of reactive oxygen species.^8^ Although Pc-LLLT is well established for antimicrobial applications, its potential as a surface conditioning modality for non-biological dental substrates remains under investigation.

The choice of resin cement is equally critical for achieving stable adhesion of GFPs to radicular dentin.^9^ Dual-cure resin cements (DCRCs) are generally preferred because their combined chemical and light-activated polymerization ensures adequate curing in the deeper regions of the root canal, where light penetration is limited. These cements can be employed with either total-etch or self-adhesive protocols; however, evidence remains inconclusive as to which cementation strategy yields superior bond strength for GFPs. 2,10

Therefore, this study aimed to evaluate the effects of different surface conditioning methods (H_2_O_2_, YFL, and Pc-LLLT) and cementation protocols on surface characterization, flexural strength, surface roughness, and push-out bond strength of GFPs bonded to radicular dentin. The null hypothesis assumed no significant differences among the experimental groups.

## METHODOLOGY

This in vitro study was conducted in accordance with the Preferred Reporting Items for Laboratory Studies in Endodontology (PRILE) guidelines for laboratory investigations in endodontology.^11^ Ethical approval was obtained from Dar al Uloom University (COD/IRB/2025/91; Dated February 15, 2025), and written informed consent was obtained from the patients. The study duration was six months.

### Specimen preparation:

Eighty single-rooted human mandibular premolars (mean root length ≈15 mm) with fully developed apices, intact roots free of caries, fractures, or resorption were selected. Teeth were stored in 0.5% thymol at 4°C for 48 hours, then decoronated at the cementoenamel junction (CEJ) using a precision sectioning machine (Microdont Technology, Germany), standardizing root length to 14 mm.

### Allocation and Surface Conditioning of GFPs:

One hundred and twenty GFPs (ParaPost Fiber White; Coltène/Whaledent, USA) were randomly allocated into four groups (n=30): Group-1 (Control)—no surface conditioning; Group-2 (H_2_O_2_)—immersion in 24% hydrogen peroxide for 20 minutes; Group-3 (YFL)—irradiation with a pulsed ytterbium fiber laser (MD-F3000, Keyence, Osaka, Japan; 1070 nm, 5 W, 60 kHz, scanning speed 50 mm/s, spot size 80 µm) in non-contact mode for 60 seconds with air cooling; Group-4 (1% Pc-LLLT)—immersion in 1% phthalocyanine solution (Santa Cruz Biotechnology, USA) for five minutes followed by LED irradiation (Fotosan 630; CMS Dental, Korea; 620–640 nm, 2–4 mW/cm²) for 60 seconds.^7^

### Surface characterization:

Two gold-sputtered specimens per group were examined under SEM (TM3030, Hitachi, Japan) at 15 kV and 1000× magnification. Surface roughness (Ra) was measured using a contact stylus profilometer (Elcometer 224/2, UK) over a 0.75 mm traverse length, with three scans per specimen (n=4). Flexural strength was determined via three-point bending (n=4) using a universal testing machine (LAM Technologies, Italy; 500 N load cell, crosshead speed 1 mm/min, span length 6 mm) and calculated using the standard formula: σ = 8FL/πd³.

### Endodontic treatment and Post cementation:

Root canals were instrumented using the WaveOne Primary reciprocating file (#25/.08, Dentsply Maillefer, Switzerland) with 3% NaOCl irrigation, obturated with the single-cone technique using AH Plus sealer, and stored at 37°C and 100% humidity for two weeks. Post spaces were prepared to a depth sufficient to accommodate the GFP using Largo Peeso reamers (#2–#4), followed by a ParaPost twist drill (Ø1.5 mm). Twenty conditioned GFPs per group were randomly assigned to two cementation subgroups (n=10): Subgroup A—conventional resin cement (RelyX ARC, 3M ESPE) with total-etch protocol (35% phosphoric acid, Single Bond adhesive); Subgroup B—self-adhesive resin cement (RelyX Universal, 3M).^12^

### Push-out Bond Strength and Failure Analysis:

Cemented roots were embedded in acrylic resin and sectioned perpendicular to the long axis into six 2-mm-thick slices representing coronal, middle, and apical root thirds. Push-out bond strength was measured using a universal testing machine (crosshead speed 0.5 mm/min) in the apical-to-coronal direction. Bond strength (MPa) was calculated as the failure load divided by the bonded surface area. Failure modes were classified as adhesive, cohesive, or mixed under stereomicroscopic examination.^13^

### Statistical analysis:

Data normality was verified using the Kolmogorov–Smirnov test. Group comparisons were performed using *Two-way ANOVA* followed by Tukey’s post hoc test (α=0.05) in SPSS version 19.0 (IBM, USA).

## RESULTS

### Ra analysis:

The Ra of the GFP varied depending on the surface-conditioning method used. The highest mean Ra value was observed in Group-2 (H_2_O_2_) (0.95 ± 0.15 µm). The lowest Ra scores were observed in Group-1 (No conditioning), which also exhibited the lowest Ra outcomes (*0.45 ± 0.10* µm). Intergroup comparison analysis revealed that Group-1 and Group-4 (Pc-LLLT) (*0.51 ± 0.11* µm) also showed no significant difference in their Ra outcomes (p> 0.05). Group-3 YFL (0.71 ± 0.14 µm), on the other hand, displayed significantly different Ra scores. (p < 0.05).

### Flexural strength assessment:

Table-II displays the FS of GFP after using different surface conditioners. The highest FS values were recorded in Group-1 (Non-conditioned) (711.12 ± 96.15 MPa). In contrast, the Group-2 (H_2_O_2_) treated samples exhibited the lowest FS (455.67 ± 55.27 MPa). Group-2 pretreatment substantially reduced the flexural strength compared to the other surface-conditioning methods. p < 0.05. Whereas, Group-1 (Non-conditioned) (711.1 ± 96.15 MPa), Group-3 YFL (664.22 ± 65.32 MPa), and Group-4 (Pc-LLLT) Group-4 (700.43 ± 78.87 MPa) showed similar FS scores. p>0.05

**Table-I:** Ra of GFP after using different surface conditioners..

Tested groups	Mean ± SD (µm)	p-value!
Group-1: No conditioning	0.45 ± 0.10^a^	< 0.05
Group-2: H_2_O_2_	0.95 ± 0.15^b^	
Group-3: YFL	0.71± 0.14^c^	
Group-4: 1% Pc-LLLT	0.51 ± 0.11^a^	

! ANOVA: Hydrogen peroxide (H_2_O_2_), Ytterbium fiber laser (YFL), Phthalocyanine (Pc), Low-level laser therapy (LLLT). Same letters in each row show no statistically significant difference (p<0.05), Post Hoc Tukey.

**Table-II:** FS of GFP after using different surface conditioners.

Tested groups	Mean ± SD (MPa)	p-value!
Group-1: No conditioning	711.12 ± 96.15 ^a^	< 0.05
Group-2: H_2_O_2_	455.67 ± 55.27 ^b^	
Group-3: YFL	664.22 ± 65.32 ^a^	
Group-4: 1% Pc-LLLT	700.43 ± 78.87 ^a^	

! ANOVA: Hydrogen peroxide (H_2_O_2_), Ytterbium fiber laser (YFL), Phthalocyanine (Pc), Low-level laser therapy (LLLT). Same letters in each row show no statistically significant difference (p<0.05), Post Hoc Tukey.

### POBS outcomes:

The highest push-out bond strengths were obtained in the cervical third of Group-2A (H_2_O_2_ + CRC) (12.53±1.54 MPa). Conversely, the lowest bond strengths were observed in the apical section of Group-4A (Pc-LLLT) + CRC (3.87 ± 0.17 MPa). Intergroup comparisons showed that Group-2A (H_2_O_2_ + CRC) (Cervical: 12.53±1.54 MPa, middle: 9.31±0.87 MPa, apical: 7.67±0.98 MPa) and Group-3A (YFL+CRC) (Cervical: 12.44±0.76 MPa, middle: 8.98±0.77 MPa, apical: 7.65±0.67 MPa) demonstrated comparable POBS of GFP to the root dentin. Likewise, Group-2B (H_2_O_2_ + SARC) (Cervical: 12.05±1.34 MPa, middle: 11.54±1.01 MPa, apical: 11.76±1.01 MPa) and Group-3B (YFL + SARC) **(**Cervical: 12.12±1.21 MPa, middle: 11.43±1.06 MPa, apical: 11.13±0.65 MPa) also demonstrated no significant difference in POBS outcomes. (p>0.05) Group-1A (No conditioning + CRC) (Cervical: 7.12±0.45 MPa, middle: 5.54±0.23 MPa, apical: 4.08±0.11MPa) and Group-4A (Pc-LLLT + CRC) (Cervical: 6.87±0.47 MPa, middle: 5.12±0.36 MPa, apical: 3.87±0.17 MPa) presented comparable scores of POBS. (p>0.05) Similarly, Group-1B (No conditioning + SARC) (Cervical: 7.85±0.22 MPa, middle: 7.96±0.63 MPa, apical: 7.43±0.21 MPa) and Group-4B (Pc (LLLT) + SARC) **(**Cervical: 7.77±0.31 MPa, middle: 7.21±0.46 MPa, apical: 6.93±0.19 MPa) also presented comparable outcomes of bond integrity. p>0.05 Intragroup comparison analysis discovered that CRC bonded groups showed a characteristic decline in bond strength from cervical to apical regions. Whereas, in SARC groups all the thirds presented comparable scores of bond strength. p>0.05.

### Failure mode assessment:

**Fig.1:**
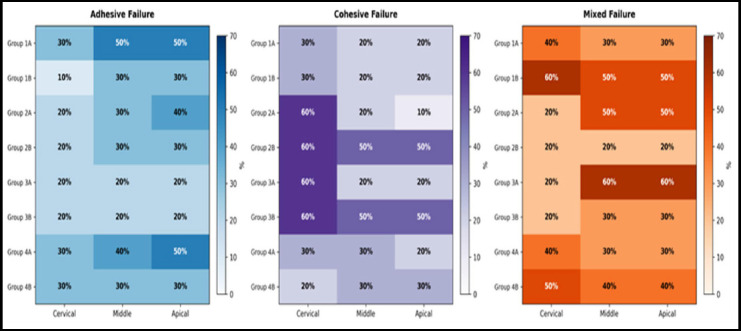
Heat map showing failure type adhesive, cohesive, and mixed patterns in coronal, middle, and apical regions among different experimental groups.

Fig.1 displays the modes of failure assessment after different groups. In group cervical sections of Groups-2A and 3A, and in all thirds of 2B and 3B, cohesive failure was most prevalent. Whereas admixed failures were observed in apical and middle sections of Group-2A and 3A, cervical sections of Group-1A and 4A, all three sections of Group-4B and 1B. Adhesive failures were observed in the middle and apical sections of 4A and 1A.

## DISCUSSION

The present investigation was predicated on the null hypothesis that no statistically significant differences would exist in the surface topography, flexural strength (FS), surface roughness (Ra), and push-out bond strength (POBS) of glass fiber posts (GFPs) conditioned with H_2_O_2_, ytterbium fiber laser (YFL), and phthalocyanine-activated low-level laser therapy (Pc-LLLT) compared to the untreated control. Additionally, it was hypothesized that there would be no significant difference in POBS between conventional resin cement (CRC) and self-adhesive resin cement (SARC). Based on the findings, the first hypothesis was partially accepted, while the second was entirely rejected. Bond strength was evaluated using the push-out test, which applies shear force parallel to the long axis of the post, closely simulating the clinical scenario of post dislodgement. 14

**Table-III:** POBS of GFP to radicular dentin after using different surface conditioners.

Tested groups	Mean ± SD (MPa) Cervical	Mean ± SD (MPa) Middle	Mean ± SD (MPa) Apical	p-value!
Group-1A: No conditioning + CRC	7.12±0.45 ^d,A^	5.54±0.23 ^d,B^	4.08±0.11 ^d,B^	< 0.05
Group-1B: No conditioning + SARC	7.85±0.22^c, A^	7.96±0.63 ^c,A^	7.43±0.21 ^c,A^	
Group-2A: H_2_O_2_ + CRC	12.53±1.54 ^a,A^	9.31±0.87 ^a,B^	7.67±0.98 ^a,B^	
Group-2B: H_2_O_2_ + SARC	12.05±1.34 ^b,A^	11.54±0.98 ^b,A^	11.76±1.01 ^b,A^	
Group-3A: YFL + CRC	12.44±0.76 ^a,A^	8.98±0.77 ^a,B^	7.65±0.67 ^a,B^	
Group-3B: YFL + SARC	12.12±1.21 ^b,A^	11.43±1.06 ^b,A^	11.13±0.65 ^b,A^	
Group-4A: 1%Pc-LLLT + CRC	6.87±0.47 ^d,A^	5.12±0.36 ^d,B^	3.87±0.17 ^d,B^	
Group-4B: 1%Pc-LLLT + SARC	7.77±0.31 ^c,A^	7.21±0.46 ^c,A^	6.93±0.19 ^c,A^	

! ANOVA: Hydrogen peroxide (H_2_O_2_), Ytterbium fiber laser (YFL), Phthalocyanine (Pc) low-level laser therapy (LLLT), Self-adhesive resin cement (SARC), Conventional resin cement (CRC). Data with Different superscript lower-case alphabets denote statistically significant differences within the same column (p < 0.05). Data with different upper-case letters denote significant differences within each row (p < 0.05), Post Hoc Tukey.

Surface pretreatment with H_2_O_2_ yielded significantly higher R_a_ and POBS values compared to the control, consistent with findings reported by AlRefeai et al.^10^ H_2_O_2_ acts by oxidatively degrading the epoxy resin matrix, thereby exposing the embedded glass fibers and increasing the effective bonding surface area available for micromechanical interlocking with resin cement.^10^ SEM examination corroborated these findings, revealing a partially degraded matrix with intact fibers and an irregular interfibrillar network. However, H_2_O_2_-treated specimens exhibited significantly lower FS than all other groups, likely attributable to the partial destruction of epoxy resin crosslinks and excessive fiber exposure, both of which compromise the structural integrity of the post.^15^

YFL-treated specimens demonstrated lower R_a_ values than H_2_O_2_-treated posts, yet achieved comparable POBS. This is the first study to evaluate the effect of YFL on the surface characteristics and bond strength of GFPs. Basunbul et al. reported that YFL irradiation improved the roughness and shear bond strength of zirconia to resin cement, supporting the potential of near-infrared laser modification for enhancing adhesion.^16^ The comparable bond strength despite lower Ra can be explained by the findings of Unal et al., who demonstrated that higher wettability and surface energy are more critical determinants of adhesion than Ra alone, as excessive roughness does not necessarily translate to improved wettability.^17^

Importantly, YFL irradiation did not significantly compromise the FS of GFPs, consistent with Kirmali et al., who reported that low-power laser irradiation delivers insufficient energy to induce major microstructural changes or thermal damage.^18^ Pc-LLLT, investigated here for the first time as a surface conditioner for GFPs, yielded unsatisfactory results. This can be attributed to the hydrophobic and cationic nature of phthalocyanine, which may induce electrostatic repulsion with negatively charged adhesive components.^19^ Furthermore, light-activated generation of reactive oxygen species and free radicals may interfere with resin polymerization, contributing to diminished adhesion.^8^ SEM analysis confirmed the absence of matrix degradation or fiber exposure, indicating that Pc-LLLT exerts negligible effects on GFP surface morphology, thereby explaining the preservation of FS in this group.

Regarding cementation strategy, SARC demonstrated significantly higher overall POBS than CRC, consistent with previous findings by Aleisa et al.^20^ Notably, intragroup analysis revealed that cervical thirds cemented with the total-etch protocol exhibited superior bond strength, attributable to the higher density of dentinal tubules in this region.^21^ However, excessive acid etching may demineralize dentin beyond the adhesive’s infiltration capacity, potentially creating zones of weakened bonding in deeper root sections.^22^ Conversely, SARC demonstrated uniformly high POBS across all root thirds, suggesting that tubule density is not a critical determinant for self-adhesive cementation. The relatively acidic environment of the apical root may enhance calcium availability for chemical adhesion, compensating for reduced light-curing efficacy at greater depths.^23^ Regarding failure modes, groups with the highest POBS predominantly exhibited cohesive failures, while adhesive and mixed failures were associated with lower bond strength values, consistent with Alkhudhairy et al.

### Study novelty:

This study is the first to investigate the ytterbium fiber laser (YFL) as a surface conditioning modality for glass fiber posts. Additionally, this investigation introduces phthalocyanine-mediated low-level laser therapy (Pc-LLLT) as a photodynamic surface conditioning approach for glass fiber posts, representing an entirely novel application of photodynamic therapy in endodontic post cementation. The simultaneous evaluation of two fundamentally different luting strategies — conventional resin cement with total-etch and self-adhesive resin cement — across all conditioning protocols provides the first comprehensive multifactorial dataset for these emerging surface modification techniques.

### Strengths:

In addition to existing dental literature, this study introduces the ytterbium fiber laser as a novel surface conditioning modality for glass fiber posts, achieving an optimal balance between enhanced surface roughness and preserved flexural strength, unlike hydrogen peroxide, which compromised mechanical integrity. It provides the first evidence on phthalocyanine-mediated photodynamic therapy as a post-surface modifier, expanding PDT applications into adhesive endodontics. The findings demonstrate that self-adhesive resin cement outperforms conventional resin cement, supporting simplified cementation protocols in confined root canal spaces. Importantly, an inverse relationship between surface roughness and flexural strength was established, cautioning against excessive surface modification of fiber-reinforced composites. Additionally, region-specific push-out bond strength analysis confirmed a progressive apical decline in bonding, providing evidence-based guidance for optimizing conditioning and cementation strategies across all root canal regions.

### Limitations:

The findings of this study should be interpreted within the limitations inherent to in vitro investigations. Specimens were not shielded from ambient light during sectioning or push-out testing, which may have affected resin cement polymerization. Furthermore, only a single set of laser parameters and a single H_2_O_2_ concentration were evaluated, which may limit the generalizability of the results.

## CONCLUSION

A yttrium fiber laser demonstrated acceptable surface roughness, flexural strength, and push-out bond strength of the glass fiber post. Self-adhesive resin cement outperformed conventional luting cement.

### Recommendations:

Future studies employing varied parameters and thermocycling aging protocols are warranted.
